# Iron deficiency and dementia risk: evidence from the Swedish population-based cohort study AMORIS

**DOI:** 10.1186/s12916-026-04839-3

**Published:** 2026-04-08

**Authors:** Mozhu Ding, Alexandra Wennberg, Stina Ek, Niklas Hammar, Katharina Schmidt-Mende, Karin Modig

**Affiliations:** 1 Unit of Epidemiology, Institute of Environmental Medicine, Karolinska Institutet, Stockholm, Sweden; 2 Academic Primary Health Care Centre, Stockholm, Stockholm Region Sweden; 3 Division of Family Medicine and Primary Care, Department of Neurobiology, Care Sciences and Society, Karolinska Institutet, Huddinge, Sweden

**Keywords:** Iron deficiency, Dementia diagnosis, Population-based study

## Abstract

**Background:**

Iron deficiency (ID) is proposed to be involved in cognitive aging and dementia; however, empirical data is lacking to support this hypothesis. We examined the association between absolute and functional ID and incident dementia diagnosis.

**Methods:**

Data from 70,935 individuals aged ≥ 50 years from the Swedish AMORIS cohort, who had blood measurements indicative of iron status between 1985–1996, were used. Participants were followed for incident dementia diagnosis recorded in National Patient Register (inpatient and specialist outpatient) and Prescribed Drug Register (dispensed anti-dementia drugs) for up to 15 years. Two exposure groups were defined: absolute iron deficiency (serum ferritin < 30 ug/L) and functional iron deficiency (transferrin saturation < 20% and serum ferritin ≥ 30 ug/L). The reference group included individuals with measurements in the normal range of hemoglobin, serum iron, and total iron binding capacity.

**Results:**

A total of 4,994 individuals received a dementia diagnosis over a mean of 12.6 years. Compared with the reference group, absolute and functional ID was associated with increased dementia diagnosis (adjusted hazard ratio (HR) = 1.24, 95% confidence interval (CI): 1.18–1.42; HR = 1.21, 95% CI: 1.05–1.39, respectively), after adjusting for age, sex, education, and comorbidities. The associations were essentially consistent across subgroups, and when further adjusting for kidney function, body mass index, and smoking in subsamples.

**Conclusions:**

Even though absolute and functional iron deficiency has different underlying mechanisms, we found that both conditions are associated with an increased risk of dementia. Considering that iron deficiency is a pervasive but often neglected health issue in older adults, resolving iron deficiency may be relevant for dementia prevention.

**Supplementary Information:**

The online version contains supplementary material available at 10.1186/s12916-026-04839-3.

## Background

Approximately 55 million individuals worldwide currently live with dementia and this number is projected to increase to 139 million by 2050 [1]. The global economic cost of dementia was approximately 1.3 trillion USD in 2019 including healthcare expenditures and reduced quality of life [2]. Given that no cure currently exists for dementia, studying its modifiable risk factors has been a top research priority for understanding the etiology of dementia and identifying preventive targets [3].

Iron is a key element in maintaining normal physiological processes such as oxygen transport, DNA synthesis, and energy production. In the brain, iron is critical for maintaining the high metabolic needs of neurons [4]. Older age is associated with higher risk of iron deficiency, with a reported prevalence between 10–50% among older adults [5–7]. The hypothesis that iron deficiency may lead to cognitive decline via brain hypoxia and white matter lesions has been proposed [8–10], but empirical data to support such a hypothesis is scarce [11]. Past studies have reported an association between anemia and dementia [12–14], however, although iron deficiency can lead to some amenia cases, the majority of individuals with iron deficiency do not have concomitant anemia [15]. Previous cross-sectional studies reported a significantly lower serum level of iron among individuals with dementia [16], but the temporality of the association is unclear.

Emerging evidence suggests that iron deficiency can be categorized into absolute and functional iron deficiency [5, 7]. The former refers to severe reduction or absence of iron stores, which is commonly caused by low dietary intake of iron or internal bleeding. The latter occurs in the presence of adequate iron stores but inability to convert these stores to available iron, which is more likely to be related to comorbidity of aging and chronic inflammation. Thus, absolute and functional iron deficiency may influence dementia risk differently, given the different underlying mechanisms. Considering the high prevalence of iron deficiency and cognitive decline among older adults, more longitudinal data is needed to evaluate the association between absolute and functional iron deficiency and risk of dementia.

To this end, using data from the large population-based Swedish Apolipoprotein-Related Mortality Risk (AMORIS) cohort, this study aims to assess the longitudinal association between absolute and functional iron deficiency and risk of a dementia diagnosis.

## Methods

### Data and participants

The AMORIS cohort has a major aim to study the role of metabolic and inflammatory biomarkers in chronic diseases [17]. The cohort included 812,073 Swedish individuals of all ages from 1985 to 1996, among whom 233,584 were aged 50 years and over. These individuals had their blood tests taken either as part of a health assessment conducted at their workplaces (i.e., screening), or from blood examinations in primary or occupational health care. All laboratory tests were conducted on fresh blood samples by a single clinical laboratory, the Central Automation Laboratory (CALAB) in Stockholm, ensuring consistency. Through the Swedish unique personal identification number, individuals in the cohort were followed up with regards to vital status, inpatient and specialized outpatient diagnosis and prescribed drugs via linkages to multiple national registers, including the National Patient Register, Cause of Death Register, and Prescribed Drug Register.

Several routinely assessed biomarkers (including serum iron, total iron biding capacity (TIBC), and hemoglobin) were available for a large proportion of subjects in the cohort. However, ferritin as a core component in the determination of iron deficiency was available in comparatively few subjects and most likely measured for clinical reasons related to iron deficiency. Therefore, individuals who had measurements on ferritin were likely assessed due to actual or suspected iron deficiency even with a normal ferritin result. To resolve this issue, we identified a reference group from subjects with normal values of key iron measures (i.e., hemoglobin, iron, and TIBC), as a proxy of no iron deficiency.

**Fig. 1:**
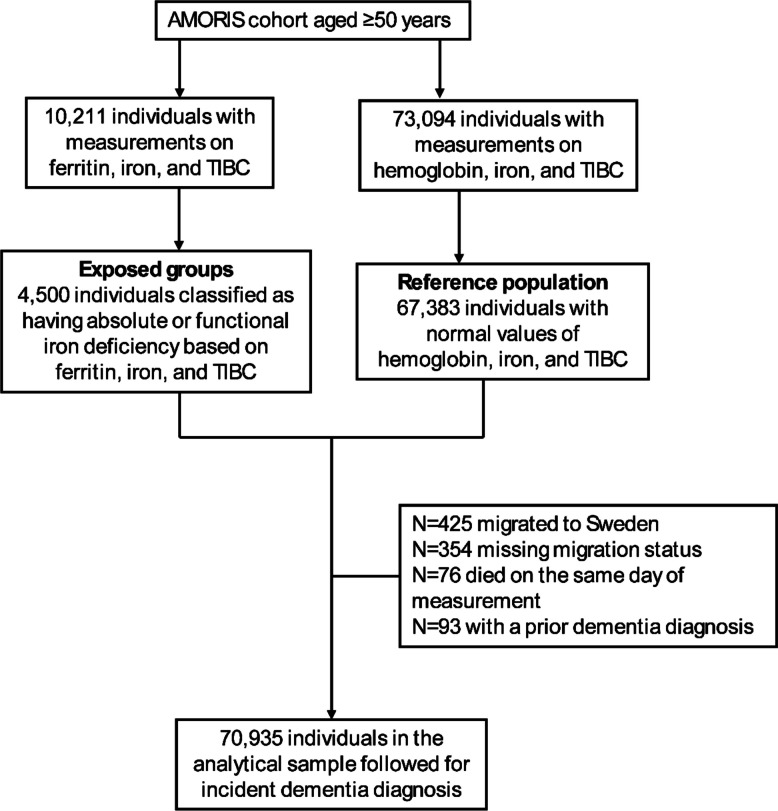
Flow chart of the study population. TIBC = total iron binding capacity

Figure 1 shows the flow chart of the study population. A total of 10,211 individuals aged 50 years and over in the AMORIS cohort had blood measurements of ferritin, iron, and TIBC on the same day. From these, we included 4,500 individuals who were classified as having absolute or functional iron deficiency based on ferritin, iron, and TIBC. A total of 73,094 individuals in the AMORIS cohort were without a ferritin measurement but had hemoglobin, iron, and TIBC measurement on the same day. From these, we identified 67,383 individuals who had normal values on hemoglobin, iron, and TIBC. We further excluded 425 individuals who migrated to Sweden because they had no prior medical records in Sweden, 354 who had missing information on migration status, 76 who died on the day of blood measurement, and 93 who had a dementia diagnosis before blood measurement. A total of 70,935 individuals were then followed for up to 15 years from the day of their first laboratory test until the date of a dementia diagnosis, date of death, emigration, or end of follow-up, whichever came first.

Lifestyle factors and kidney function can be potential confounders as they have been associated with both iron levels and risk of dementia [7, 18, 19]. Yet due to limited lifestyle data in the AMORIS cohort, we were only able to adjust for smoking and BMI as lifestyle-related factors. In order to take these risk factors into account, sensitivity analyses were performed on a smaller subset of the cohort where such information is available. A total of 68,975 individuals had concomitant measurements of estimated glomerular filtration rate (eGFR), 5,985 had measurements of BMI, and 7,930 had information on smoking status. The distribution of iron deficiency status, education, and baseline health status were similar between the full sample and the three subsamples, although individuals in with information on BMI and smoking were younger and had lower cumulative incidence of dementia (Additional file 1: Table 1).

### Assessment of iron deficiency and the reference group

Serum iron was measured via acidification with citric acid to dissociate the Fe3 + transferring complex (coefficient of variation < 5%). TIBC was assessed by adding Fe3 + to the serum. Transferring concentration in plasma determines how much iron can be bound by plasma and is expressed as TIBC. Both markers were assessed with a DAX 96, Technicon Instruments Corporation, Tarrytown, NY, USA, 1993–1996. Ferritin was assessed using Automated Chemiluminiscence Analyzer ACS:180 TM. According to previous studies, transferrin saturation was calculated as serum iron levels divided by TIBC multiplied by 100 [7]. Absolute iron deficiency was defined as serum ferritin < 30 ug/L, and functional iron deficiency was defined as transferrin saturation < 20% and serum ferritin ≥ 30 ug/L [7].

The reference group included subjects with normal values of serum iron (9–34 μmol/L), TIBC (40–78 μmol/L), and hemoglobin (≥ 130 g/L for men and ≥ 120 g/L for women), according to Swedish clinical guidelines. These biomarkers correlate with ferritin and normal values in all three can serve as a proxy for no iron deficiency [20]. Anemia was defined according to the WHO definition (< 130 g/L for men and < 120 g/L for women) [21].

### Identification of dementia diagnosis

Individuals were followed from the date of the blood test until the first date of a dementia diagnosis in in-or outpatient specialist care, first date of dispensed anti-dementia drugs, or mortality with dementia as cause of death, whichever came first. The National Patient Register (NPR) contains information on hospital discharge records from inpatient care regionally since 1964 and nationally since 1987, and data on specialized outpatient care were available nationally since 2001. Information retrieved from this register includes the dates and discharge diagnoses of each hospital visit, and all discharge diagnoses were coded according to the International Classification of Diseases Ninth and Tenth Revision (ICD-9, ICD-10). The National Cause of Death Register is a complete register of all deaths in Sweden since 1952, with ICD codes of underlying and contributing causes of deaths. In this study, dementia was identified using both diagnosis codes and dispensed anti-dementia drugs. In the NPR, dementia was identified via ICD-8 code 290, ICD-9 codes 290, 294, 331, and ICD-10 codes F00-F03, F05.1, G30, G31.0, and G31.8. ICD-9 codes 331.0 and ICD-10 codes F00 and G30 were used to identify Alzheimer’s Disease (AD), and ICD-9 codes 290.4 and ICD-10 codes F01 were used to identify vascular dementia (VaD). Dispensed anti-dementia drugs (Anatomical Therapeutic Chemical (ATC) code N06D) was assessed in the Prescribed Drug Register, which contains data on prescribed medications collected at pharmacy from July 2005 onwards. Because primary care data was not available in the current study, use of anti-dementia drugs could serve as a proxy of dementia cases that were likely diagnosed in primary care alone. Additional file 1: Fig. S1 shows the number of dementia diagnoses identified from the NPR and Prescribed Drug Register for each calendar year during the study period. Most dementia diagnoses were retrieved from the NPR.

### Covariates

Age, sex, and education data were retrieved from the Total Population Register and Longitudinal Integrated Database for Health Insurance and Labor Market Studies (LISA) register. Education was categorized according to years of formal schooling into lower than high school (≤ 9 years), high school (10–12 years), and university (≥ 13 years). Diagnosis of cardiovascular diseases prior to blood measurement was identified from the NPR for diabetes (ICD-8: 250; ICD-9: 250, 251.D; ICD-10: E10, E11, E13, E14), hypertension (ICD-8: 400–404; ICD-9: 401–405; ICD-10: I10, I13, I15), heart failure (ICD-8: 427.0, 427.1; ICD-9: 402, 404, 425, 428; ICD-10: I110, I130, I132, I27, I280, I42, I43, I50, I515, I517, I528), coronary heart disease (ICD-8: 410–414; ICD-9: 410–414; ICD-10: I20-25), atrial fibrillation (ICD-8: 427.90 and 427.92; ICD-9: 427.3; ICD-10: I48), stroke (ICD-8: 431–434; ICD-9: 431–434; ICD-10: I61, I63, I64), and TIA (ICD-8: 435; ICD-9: 435; ICD-10: G45). Charlson Comorbidity Index (CCI) was calculated following the methods developed by Ludvigsson et al., an adapted version of the CCI to be used in Swedish registers [22].

Serum creatinine was analyzed by a non-kinetic alkaline picrate method (Jaffe Method) using an AutoChemist-PRISMA during 1985–1992 and DAX-96 analyzer during1993–1996. The 2009 Chronic Kidney Disease Epidemiology Collaboration (CKD-EPI) formula was used to estimate eGFR based on serum creatinine. Smoking habits were self-reported and tobacco smoking was defined as current or former regular smoking for at least 1 year. BMI was calculated as weight (kilograms) divided by height (meters) squared.

### Statistical analysis

The association between iron deficiency and incident dementia diagnosis was examined using Cox proportional hazard regression models adjusting for age, sex, education level, and history of cardiovascular disease diagnosis, and CCI as potential confounders. Considering that iron deficiency may affect dementia risk differently among population subgroups, we further stratified the analyses by sex, age groups at baseline (50–74 and ≥ 75 years), history of cardiovascular disease diagnosis, and CCI categories (0 or ≥ 1). In both the full sample and in different subgroups, absolute and functional iron deficiency was compared against the reference group in relation to dementia risk. Moreover, because in some cases iron deficiency can result in anemia [23] and anemia has been associated with higher dementia risk [12–14], we further examined the association among people without concurrent anemia in a subset where hemoglobin measures were available (*n* = 69,448). We also performed several sensitivity analyses to test the robustness of the results. First, we conditioned on 50,305 people alive from 2001 and onwards, to account for dementia cases diagnosed exclusively in specialized outpatient care before 2001, which are not captured by our data. These cases are potentially sicker and more likely to be related to iron deficiency. Second, in the subsamples where eGFR, BMI, and smoking data were respectively available, we further adjusted for these risk factors in the model. Third, because the analytical sample was selected based on availability of iron-related biomarkers, which could potentially introduce selection bias, we performed inverse probability weighting based on baseline characteristics available in the full cohort (i.e., age, sex, education, and CCI) that could influence the likelihood of being tested due to iron related health issues. Specifically, we modeled the probability of having biomarker measurements as a function of these variables in the full cohort (*n* = 233,584) and weighed the Cox models by the inverse of this probability in the analytical sample.

Stata/SE 16.1 (StataCorp LLC, College Station, Texas, United States of America) for Windows was used for all analysis.

## Results

Among the 70,935 individuals included in the analysis, 2,241 (3.2%) were categorized as having absolute iron deficiency and 2,190 (3.1%) as functional iron deficiency. The reference group included 66,504 individuals with normal iron, TIBC, and hemoglobin. Compared to the reference group, people with functional or absolute iron deficiency were older and more likely to have a history of cardiovascular diseases and a CCI ≥ 1 (Table 1). Women constituted a much higher proportion in the absolute iron deficiency group (77.0%) than the other two groups (55.5% for reference group and 59.6% for functional iron deficiency group).

**Table 1:** Baseline characteristics of study participants by status of iron deficiency

Baseline characteristics	Reference population	Absolute iron deficiency	Functional iron deficiency
No. of subjects (%)	66,504 (93.8)	2,241 (3.2)	2,190 (3.1)
Age, mean (SD)	62.6 (9.2)	64.3 (12.1)	66.8 (11.4)
Age groups (years), n (%)			
50–74	58,378 (87.8)	1,684 (75.2)	1,590 (72.6)
≥ 75	8,123 (12.2)	557 (24.9)	600 (27.4)
Female sex, n (%)	36,874 (55.5)	1,726 (77.0)	1,305 (59.6)
Education^a^, n (%)			
Less than high school	19,738 (29.7)	786 (35.1)	751 (34.3)
High school	24,542 (36.9)	765 (34.1)	731 (33.4)
University or above	15,763 (23.7)	488 (21.8)	453 (20.7)
History of cardiovascular disease diagnosis, n (%)	6,734 (10.1)	430 (19.2)	452 (20.6)
Coronary heart disease	3,312 (5.0)	211 (9.4)	204 (9.3)
Heart failure	1,061 (1.6)	137 (6.1)	152 (6.9)
Atrial fibrillation	1,347 (2.0)	80 (3.6)	107 (4.9)
Hypertension	1,832 (2.8)	131 (5.9)	146 (6.7)
Diabetes	1,012 (1.5)	68 (3.0)	70 (3.2)
Stroke	781 (1.2)	67 (3.0)	75 (3.4)
Charlson Comorbidity Index			
Mean (SD)	0.4 (0.9)	0.5 (0.9)	0.5 (0.9)
0, n (%)	49,675 (74.7)	1,492 (66.6)	1,397 (63.8)
≥ 1, n (%)	16,829 (25.3)	749 (33.4)	793 (36.2)
Incident dementia diagnosis, n (%)	4,579 (6,9)	212 (9.5)	203 (9.3)

^a^Missing in education accounts for 9.5%. SD = standard deviation

Over a mean follow-up of 12.6 years (SD 4.1), 4,994 individuals (7.0%) received a new dementia diagnosis. Table 2 shows the absolute and relative risk of dementia diagnosis by iron deficiency status in the total cohort and in different subgroups. Men with absolute iron deficiency had higher incidence rate (IR) of dementia (IR = 10.6, 95% confidence interval (CI): 8.1–13.9) than men with functional iron deficiency (IR = 6.8, 95% CI: 5.2–8.7), while the opposite pattern was observed for women. Moreover, in age group ≥ 75 years, people with functional iron deficiency had higher IR of dementia (IR = 32.4, 95% CI: 26.9–38.9) than those with absolute iron deficiency. In people with comorbidities (CCI ≥ 1), IR of dementia was higher in absolute than in functional iron deficiency. No substantial differences in dementia incidence were observed in other subgroups.

**Table 2:** Hazard ratios and 95% confidence interval for the association between iron deficiency and dementia diagnosis, in the total cohort and stratified by age groups, sex, and history of cardiovascular disease diagnosis

	**Reference population**	**Absolute iron deficiency**	**Functional iron deficiency**
**Total cohort**			
n/N	4,579/66,504	212/2,241	203/2,190
IR per 1000 PY (95% CI)	5.4 (5.2–5.6)	8.3 (7.3–9.5)	9.1 (7.9–10.5)
HR^a^ (95% CI)	Reference (1.00)	1.24 (1.08–1.43)^b^	1.21 (1.05–1.40)^b^
**Men**			
n/N	1512/29,630	52/515	59/885
IR per 1000 PY (95% CI)	4.0 (3.8–4.2)	10.6 (8.1–13.9)	6.8 (5.2–8.7)
HR^a^ (95% CI)	Reference (1.00)	1.39 (1.05–1.84)^b^	1.25 (0.96–1.62)
**Women**			
n/N	3,067/36,874	160/1,726	144/1,305
IR per 1000 PY (95% CI)	6.5 (6.2–6.7)	7.8 (6.6–9.1)	10.6 (9.0–12.5)
HR^a^ (95% CI)	Reference (1.00)	1.20 (1.02–1.41)^b^	1.20 (1.01–1.42)^b^
**Age group 50–74 years**			
n/N	29,34/58,378	107/1,684	90/1,590
IR per 1000 PY (95% CI)	3.7 (3.6–3.9)	4.9 (4.1–6.0)	4.8 (3.9–5.9)
HR^a^ (95% CI)	Reference (1.00)	1.50 (1.24–1.83)^b^	1.19 (0.97–1.47)
**Age group ≥ 75 years**			
n/N	1,645/8,126	105/557	113/600
IR per 1000 PY (95% CI)	23.7 (22.6–24.9)	27.7 (22.9–33.5)	32.4 (26.9–38.9)
HR^a^ (95% CI)	Reference (1.00)	1.18 (0.97–1.44)	1.40 (1.16–1.70)^b^
**With a history of CVD diagnosis**			
n/N	675/6,734	55/430	59/452
IR per 1000 PY (95% CI)	9.9 (9.1–10.6)	16.4 (12.5–21.4)	18.6 (14.5–24.1)
HR^a^ (95% CI)	Reference (1.00)	1.34 (1.01–1.76)^b^	1.49 (1.14–1.95)^b^
**Without a history of CVD diagnosis**			
n/N	3,904/59,770	157/1,811	144/1,738
IR per 1000 PY (95% CI)	5.0 (4.8–5.2)	7.1 (6.1–8.3)	7.5 (6.4–8.9)
HR^a^ (95% CI)	Reference (1.00)	1.22 (1.04–1.43)^b^	1.14 (0.96–1.34)
**Having a CCI of 0**			
n/N	2,512/43,297	106/1,345	106/1,157
IR per 1000 PY (95% CI)	4.1 (3.9–4.2)	5.7 (4.7–6.9)	6.8 (5.6–8.3)
HR^a^ (95% CI)	Reference (1.00)	1.07 (0.89–1.29)	1.19 (1.00–1.62)^b^
**Having a CCI ≥ 1**			
n/N	799/11,562	72/488	49/456
IR per 1000 PY (95% CI)	5.0 (4.7–5.4)	12.5 (9.9–15.7)	5.5 (6.7–11.8)
HR^a^ (95% CI)	Reference (1.00)	1.54 (1.24–1.91)^b^	1.28 (1.01–1.62)^b^

^a^All hazard ratios are adjusted for age, sex, education level, history of cardiovascular diagnosis, and Charlson Comorbidity Index. *n* number of incident dementia diagnosis, *N* number of subjects, *IR* incidence rate, *HR* hazard ratio, *CI* confidence interval, *PY* person-years, *CCI* Charlson Comorbidity Index

^b^*p* < 0.05

With regards to relative risk, absolute and functional iron deficiency were both associated with a higher risk of dementia diagnosis after adjusting for age, sex, education, history of cardiovascular disease diagnosis, and CCI (hazard ratio (HR) = 1.24, 95% confidence interval (CI): 1.08–1.43; HR = 1.21, 95% CI: 1.05–1.40, respectively). The associations were essentially consistent when stratified by sex, age groups, cardiovascular disease diagnosis, and CCI categories. When further investigating dementia subtypes, 32.4% and 13.8% of all dementia diagnoses were AD and VaD respectively, and 53.8% were unspecified dementia. The association of absolute and functional iron deficiency with risk of AD was similar to the main analysis (HR = 1.36, 95% CI: 1.02–1.81; HR = 1.35, 95% CI: 1.02–1.81), and the results for VaD attenuated (HR = 0.99, 95% CI: 0.65–1.50; HR = 1.10, 95% CI: 0.74–1.63). Further, when stratifying by anemia status, 56% of people with absolute iron deficiency and 71% of people with functional iron deficiency did not have concurrent anemia. Anemia was statically significantly associated with a higher risk of dementia in the current study (adjusted HR = 1.23, 95% CI: 1.01–1.53). Among people without anemia, the association for absolute iron deficiency remained (HR = 1.46, 95% CI: 1.19–1.79) but attenuated for functional iron deficiency (HR = 1.08, 95% CI: 0.88–1.33).

In the sensitivity analysis conditioning on individuals alive from 2001 onwards, to reduce the influence of missed dementia diagnosis before the availability of specialized outpatient data in 2001, the association between absolute and functional iron deficiency and dementia risk was consistent with the main analysis, both in the total cohort and in subgroups (Additional file 1: Table 2). Moreover, in subsamples where data on eGFR, BMI, and smoking were respectively available, mean eGFR was significantly lower and the proportion of smokers significantly higher among those with functional iron deficiency compared to the absolute iron deficiency and reference population (p < 0.05) (Additional file 1: Table 3). However, there was no substantial difference in mean BMI by iron deficiency status. Further adjusting the model for these factors in their respective samples did not alter the point estimates substantially although the association for functional iron deficiency was statistically insignificant in the eGFR and BMI subsamples. Finally, in the inverse probability weighting analysis, weighted results were very similar to the unweighted results (Additional file 1: Table 4), suggesting that the observed association is unlikely to be explained by selection into the analytical sample due to differential age, sex, education, and comorbidity burden at baseline.

## Discussion

This large population-based study found a higher risk of dementia in older adults with absolute or functional iron deficiency compared to individuals with normal values of iron, TIBC, and hemoglobin. The association was largely consistent across sexes, age groups, and among individuals with and without comorbidities, or when further adjusting for eGFR, BMI, or smoking. These findings suggest that both absolute and functional iron deficiency in circulating blood may contribute to the pathogenesis of dementia.

To the best of our knowledge, this is the first longitudinal study to investigate the association between iron deficiency and the risk of dementia. Previous evidence on this topic has primarily come from cross-sectional studies. For example, a recent meta-analysis of cross-sectional studies, including 2,174 individuals with dementia and 2,931 cognitively healthy controls, reported lower serum iron levels in people with dementia, while no differences were observed for serum ferritin or other iron markers [16]. Importantly, as cross-sectional designs cannot account for reverse causality, the observed association in these studies may reflect low iron levels as a consequence of dementia, particularly given the high prevalence of malnutrition in this population [24]. In contrast, our longitudinal findings argue against reverse causality, demonstrating that iron deficiency measured a decade before dementia onset is associated with an increased risk of dementia. Moreover, a few prospective cohort studies reported a higher risk of dementia among women with very low dietary iron intake, [25] or people with anemia defined by low hemoglobin levels in blood [12–14]. One of the proposed mechanisms in these studies is brain hypoxia resulting from iron deficiency associated with anemia, which may subsequently lead to neurodegeneration. A major distinction between our study and previous research, beyond the longitudinal design, lies in the definition of iron deficiency. In this study, we defined iron deficiency based on a combination of serum iron, ferritin, and TIBC. This is a more clinically robust measure of iron deficiency compared to prior studies which have predominantly used serum iron or hemoglobin as proxies. Serum iron in isolation does not provide a reliable measure of iron stores, because it fluctuates significantly during the day and is sensitive to dietary intake [26]. Moreover, previous research has shown that iron deficiency can occur independently of anemia [27, 28]. In fact, findings from community-dwelling older adults in Europe indicated that 89% of individuals with iron deficiency did not have anemia. [15] Our data are in line with these previous studies, and the association between iron deficiency and dementia risk persisted even among people without anemia.

Our study also contributes to the literature by examining dementia risk in individuals with functional iron deficiency, which can occur even in the presence of adequate iron stores. Functional iron deficiency is reported to be more prevalent than absolute iron deficiency among people aged ≥ 50 years [7], and is associated with systemic inflammation and cardiovascular comorbidities—both of which have been linked to an increased risk of dementia [29–31]. Indeed, in our study, functional iron deficiency appeared to be more strongly associated with elevated dementia risk among those aged ≥ 75 years or those with a history of CVD or other comorbidities. Moreover, a recent study found that functional iron deficiency was more strongly associated with obesity than with any other comorbidities or lifestyle factors [7]. Because of data restrictions we could not study the impact of lifestyle-related factors in the full sample, but in smaller subsamples. We found that BMI did not differ substantially by iron deficiency status, while smoking was more prevalent among those with functional iron deficiency. Nevertheless, the associations between iron deficiency and dementia were similar when adjusting for BMI and smoking, albeit statistically insignificant for functional iron deficiency in the BMI subsample, possibly due to lack of power.

It is important to note that iron levels in blood may not always reflect iron metabolism in the brain, as transfer of iron from peripheral circulation to the brain is tightly regulated by the brain blood barrier to prevent iron overload in the brain [32]. At the same time, the brain appears to have a greater capacity than other organs to retain iron and resist nutritional iron deficiency [4]. Therefore, even though we report an association between blood iron deficiency and dementia risk, the role of iron levels in the brain or cerebrospinal fluid in dementia pathology remains to be further explored. In fact, in vivo and post-mortem studies [33, 34] have led to hypotheses that iron deposits in the brain may be associated with the aggregation of amyloid beta and tau which are key contributors in the pathophysiology of Alzheimer’s disease [35].

This study utilized a large, population-based longitudinal cohort to examine the association between both absolute and functional iron deficiency and the risk of dementia. Moreover, the fact that all biomarkers were analyzed in the same laboratory for all individuals ensured consistency in iron deficiency assessment. However, the findings should be interpreted in light of several limitations. First, dementia cases were identified using diagnoses through specialist care diagnoses, and from 2005 and onwards, also dispensed anti-dementia drugs. While this approach ensures nation-wide coverage, it lacks sensitivity for detecting all dementia cases, particularly those with mild or moderate dementia diagnosed solely in primary care. This likely introduced nondifferential misclassification, which would attenuate observed associations by affecting individuals with and without iron deficiency equally. However, some degree of differential misclassification cannot be entirely ruled out and may have biased the true effect. Second, because in the clinical setting subtypes of dementia are often not further investigated and instead coded as unspecified, the association for dementia subtypes in our study are likely diluted. Moreover, future studies could benefit from utilizing more sensitive measures of cognition, such as standardized cognitive test scores or longitudinal cognitive trajectories. Third, there may be unmeasured confounding not captured by our data. Because lifestyle information in the AMORIS cohort was limited, we were only able to adjust for BMI and smoking in the subsamples. More studies are warranted to validate our results and consider other lifestyle factors. Fourth, although we performed inverse probability weight analysis to account for potential selection bias, residual selection due to unmeasured determinants of biomarker testing cannot be fully excluded. Finally, although iron deficiency is common among older adults in high-income countries [14], it is even more prevalent in low- and middle-income countries, where its implications for cognitive health remain underexplored [36]. Given geographical differences in the distribution of both iron deficiency and dementia risk factors and the projected rise in dementia incidence in low- and middle-income countries [37], the association observed in this Swedish cohort may not be directly generalizable to other populations. Further studies are warranted in diverse settings to assess the consistency and contextual relevance of these findings.

## Conclusions

Even though absolute and functional iron deficiency have different underlying mechanisms, we found both conditions are associated with an increased risk of dementia. Further research is needed to confirm these findings and explore the mechanisms linking iron deficiency to dementia pathology in the brain. Considering that iron deficiency is a pervasive but often neglected health issue in older adults, resolving iron deficiency may be relevant for dementia prevention.

## Supplementary Information

Additional file 1.

## Data Availability

The datasets analyzed during the current study are not publicly available due to the General Data Protection Regulation in Sweden. Access to the data and the codes for data analyses can be permitted to external researchers after ethical vetting and establishment of a collaboration agreement. Contact the corresponding author for questions about data sharing (MD).

## Abbreviations

AMORIS: Swedish Apolipoprotein-Related Mortality Risk
ICD: International Classification of Diseases
ATC: Anatomical Therapeutic Chemical
HR: Hazard ratio
CI: Confidence interval
CCI: Charlson comorbidity index
BMI: Body mass index
eGFR: Estimated glomerular filtration rate
IR: Incidence rate
TIBC: Total iron binding capacity
SD: Standard deviation
NPR: National Patient Register
