# Supplementary material for: Iron deficiency and dementia risk: evidence from the Swedish population-based cohort study AMORIS
Source: BMC Med. 2026 Apr 8;24:226. doi: 10.1186/s12916-026-04839-3 (PMC13067417; doi:10.1186/s12916-026-04839-3)
Supplement: Supplementary file 1 — Additional file 1. [file 12916_2026_4839_MOESM1_ESM.docx]

**
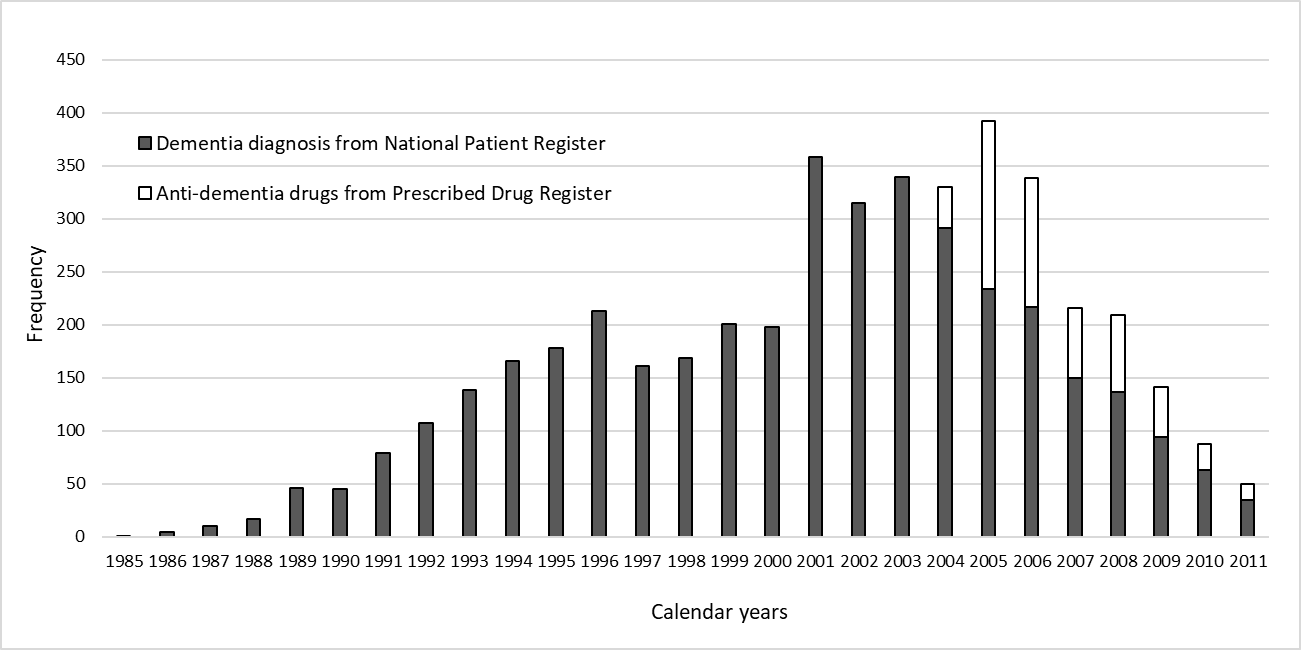
**

**Figure S1.** Distribution of number dementia diagnosis identified from National Patient Register or Prescribed Drug Register by calendar year during the study period.

**Table 1**. Baseline characteristics by status of iron deficiency in the full and subsamples.

| **Baseline characteristics** | Full sample (n=70,935) | Subsample for eGFR (n=68,975) | Subsample for BMI (n=5,985) | Subsample for smoking (n=7,930) |
| --- | --- | --- | --- | --- |
| Iron deficiency status, n (%) |  |  |  |  |
| Reference population | 66,504 (93.8) | 65,684 (95.2) | 5,544 (93.0) | 7,547 (95.1) |
| Absolute iron deficiency | 2,241 (3.2) | 1,539 (2.2) | 212 (3.6) | 187 (2.4) |
| Functional iron deficiency | 2,190 (3.1) | 1,752 (2.6) | 202 (3.4) | 196 (2.5) |
| Age, mean (SD) | 62.8 (9.4) | 62.7 (9.4) | 59.6 (7.8) | 58.2 (6.3) |
| Age groups (years), n (%) |  |  |  |  |
| 50-74 | 61,652 (86.9) | 60,118 (87.2) | 5,602 (94.0) | 7,790 (98.2) |
| ≥75 | 9,283 (13.1) | 8,857 (12.8) | 356 (6.0) | 140 (1.8) |
| Female sex, n (%) | 39,905 (56.3) | 38,557 (55.9) | 3,310 (55.6) | 3,721 (46.9) |
| Education^a^, n (%) |  |  |  |  |
| Less than high school | 21,275 (30.0) | 20,642 (29.9) | 1,939 (32.5) | 2,570 (32.4) |
| High school | 26,038 (36.7) | 25,359 (36.8) | 2,386 (40.1) | 3,271 (41.3) |
| University or above | 16,704 (23.6) | 16,222 (23.5) | 1,407 (23.6) | 1,910 (24.1) |
| History of cardiovascular disease diagnosis, n (%) | 7,616 (10.7) | 7,329 (10.6) | 498 (8.4) | 937 (11.8) |
| Charlson Comorbidity Index |  |  |  |  |
| Mean (SD) | 0.5 (1.0) | 0.5 (1.0) | 0.4 (1.0) | 0.4 (0.9) |
| 0, n (%) | 52,564 (74.1) | 51,205 (74.2) | 4,625 (77.6) | 6,091 (76.8) |
| ≥1, n (%) | 18,371 (25.9) | 17,770 (25.8) | 1,333 (22.4) | 1,839 (23.2) |
| Incident dementia diagnosis, n (%) | 4,994 (7.0) | 4,816 (6.9) | 287 (4.8) | 197 (2.5) |

^a^Missing in education accounts for 9.5%. SD=standard deviation.

**Table 2**. Hazard ratios and 95% confidence interval for the association between iron deficiency and dementia diagnosis, conditioning on individuals alive from 2001 onwards (n= 58,305)

|  | **Reference population** | **Absolute iron deficiency** | **Functional iron deficiency** |
| --- | --- | --- | --- |
| **Total sample** | Reference (1.00) | 1.43 (1.23-1.66)^a^ | 1.28 (1.09-1.51)^a^ |
| **Men** | Reference (1.00) | 1.56 (1.13-2.16)^a^ | 1.33 (0.98-1.80) |
| **Women** | Reference (1.00) | 1.41 (1.18-1.67)^a^ | 1.26 (1.04-1.53)^a^ |
| **Age group 50-74** | Reference (1.00) | 1.70 (1.39-2.07)^a^ | 1.27 (1.01-1.59)^a^ |
| **Age group ≥75** | Reference (1.00) | 1.28 (1.01-1.62)^a^ | 1.40 (1.10-1.77)^a^ |
| **With a history of CVD diagnosis** | Reference (1.00) | 1.45 (1.04-2.01)^a^ | 1.40 (1.00-1.97)^a^ |
| **Without a history of CVD diagnosis** | Reference (1.00) | 1.43 (1.21-1.70)^a^ | 1.25 (1.04-1.51)^a^ |
| **CCI =0** | Reference (1.00) | 1.27 (1.04-1.54)^a^ | 1.32 (1.09-1.60)^a^ |
| **CCI ≥1** | Reference (1.00) | 1.73 (1.35-2.21)^a^ | 1.22 (0.91-1.63) |

All hazard ratios are adjusted for age, sex, education level, and history of cardiovascular diagnosis, and Charlson Comorbidity Index. ^a^p<0.05

**Table 3**. Hazard ratios and 95% confidence interval for the association between iron deficiency and dementia diagnosis, conditioning on individuals who had concurrent measurements on eGFR, body mass index, and smoking, respectively.

|  | **Reference population** | **Absolute iron deficiency** | **Functional iron deficiency** |
| --- | --- | --- | --- |
| **Subsample for eGFR (n=68,975)** |  |  |  |
| eGFR, mean (SD) | 77.1 (14.3) | 76.8 (17.0) | 74.6 (18.5) |
| Model 1 | Reference (1.00) | 1.30 (1.10-1.53)^a^ | 1.16 (0.99-1.37) |
| Model 1 + eGFR | Reference (1.00) | 1.31 (1.13-1.55)^a^ | 1.15 (0.98-1.36) |
| **Subsample for BMI (n=5,985)** |  |  |  |
| BMI, mean (SD) | 25.1 (3.8) | 24.7 (3.6) | 25.0 (3.6) |
| Model 1 | Reference (1.00) | 1.73 (1.02-2.93)^a^ | 1.58 (0.96-2.62) |
| Model 1 + BMI | Reference (1.00) | 1.75 (1.03-2.96)^a^ | 1.58 (0.95-2.62) |
| **Subsample for smoking (n=7,930)** |  |  |  |
| Ever smoking, n (%) | 1,195 (15.8) | 22 (11.8) | 47 (23.9) |
| Model 1 | Reference (1.00) | 2.64 (1.49-4.68)^a^ | 2.08 (1.06-4.07)^a^ |
| Model 1 + eGFR | Reference (1.00) | 2.65 (1.49-4.68)^a^ | 2.10 (1.07-4.11)^a^ |

Model 1 is adjusted for age, sex, education level, history of cardiovascular disease diagnosis, and Charlson Comorbidity Index. ^a^p<0.05

**Table 4**. Inverse probability weighted hazard ratios and 95% confidence interval for the association between iron deficiency and dementia diagnosis.

|  | **Reference population** | **Absolute iron deficiency** | **Functional iron deficiency** |
| --- | --- | --- | --- |
| **Total sample** | Reference (1.00) | 1.20 (1.04-1.39)^a^ | 1.21 (1.04-1.41)^a^ |
| **Men** | Reference (1.00) | 1.38 (1.00-1.79)^a^ | 1.24 (0.94-1.65) |
| **Women** | Reference (1.00) | 1.16 (0.98-1.37) | 1.19 (1.00-1.42)^a^ |
| **Age group 50-74** | Reference (1.00) | 1.44 (1.18-1.76)^a^ | 1.21 (0.97-1.51) |
| **Age group ≥75** | Reference (1.00) | 1.14 (0.93-1.40) | 1.38 (1.13-1.69)^a^ |
| **With a history of CVD diagnosis** | Reference (1.00) | 1.19 (1.01-1.41)^a^ | 1.36 (1.14-1.62)^a^ |
| **Without a history of CVD diagnosis** | Reference (1.00) | 1.39 (1.04-1.85)^a^ | 1.61 (1.21-2.13)^a^ |
| **CCI =0** | Reference (1.00) | 1.07 (0.88-1.29)^a^ | 1.36 (1.13-1.64)^a^ |
| **CCI ≥1** | Reference (1.00) | 1.71 (1.37-2.13)^a^ | 1.56 (1.23-1.99)^a^ |

All hazard ratios are adjusted for age, sex, education level, and history of cardiovascular diagnosis, and Charlson Comorbidity Index. ^a^p<0.05
